# Aortic valve calcification volumes and chronic brain infarctions in patients undergoing transcatheter aortic valve implantation

**DOI:** 10.1007/s10554-019-01663-0

**Published:** 2019-07-16

**Authors:** Wieneke Vlastra, Thomas P. W. van den Boogert, Thomas Krommenhoek, Anne-Sophie G. T. Bronzwaer, Henk J. M. M. Mutsaerts, Hakim C. Achterberg, Esther E. Bron, Wiro J. Niessen, Charles B. L. M. Majoie, Aart J. Nederveen, Jan Baan, Johannes J. van Lieshout, Jan J. Piek, R. Nils Planken, José P. S. Henriques, Ronak Delewi

**Affiliations:** 1 Department of Clinical and Experimental Cardiology, Amsterdam Cardiovascular Sciences, Amsterdam UMC, University of Amsterdam, Heart Center, Amsterdam, The Netherlands; 2 Department of Internal Medicine, Amsterdam UMC, University of Amsterdam, Amsterdam, The Netherlands; 3 Laboratory for Clinical Cardiovascular Physiology, Center for Heart Failure Research, Academic Medical Center, University of Amsterdam, Amsterdam, The Netherlands; 4 Department of Radiology and Nuclear Medicine, Amsterdam University Medical Centers, Location AMC and VUmc, University of Amsterdam, Amsterdam, The Netherlands; 5 Department of Radiology & Nuclear Medicine, Biomedical Imaging Group Rotterdam, Erasmus MC-University Medical Center Rotterdam, Rotterdam, The Netherlands; 6 MRC/Arthritis Research UK Centre for Musculoskeletal Ageing Research, School of Life Sciences, The Medical School, University of Nottingham Medical School, Queen’s Medical Centre, Nottingham, UK

**Keywords:** Transcatheter aortic valve implantation, Transcatheter aortic valve replacement, Silent brain infarctions, White matter hyperintensities, Cerebral embolizations

## Abstract

Chronic silent brain infarctions, detected as new white matter hyperintensities on magnetic resonance imaging (MRI) following transcatheter aortic valve implantation (TAVI), are associated with long-term cognitive deterioration. This is the first study to investigate to which extent the calcification volume of the native aortic valve (AV) measured with cardiac computed tomography angiography (CTA) predicts the increase in chronic white matter hyperintensity volume after TAVI. A total of 36 patients (79 ± 5 years, median EuroSCORE II 1.9%, Q_1_–Q_3_ 1.5–3.4%) with severe AV stenosis underwent fluid attenuation inversion recovery (FLAIR) MRI < 24 h prior to TAVI and at 3 months follow-up for assessment of cerebral white matter hyperintensity volume (mL). Calcification volumes (mm^3^) of the AV, aortic arch, landing zone and left ventricle were measured on the CTA pre-TAVI. The largest calcification volumes were found in the AV (median 692 mm^3^) and aortic arch (median 633 mm^3^), with a large variation between patients (Q_1_–Q_3_ 482–1297 mm^3^ and 213–1727 mm^3^, respectively). The white matter hyperintensity volume increased in 72% of the patients. In these patients the median volume increase was of 1.1 mL (Q_1_–Q_3_ 0.3–4.6 mL), corresponding with a 27% increase from baseline (Q_1_–Q_3_ 7–104%). The calcification volume in the AV predicted the increase of white matter hyperintensity volume (Δ%), with a 35% increase of white matter hyperintensity volume, per 100 mm^3^ of AV calcification volume (SE 8.5, p < 0.001). The calcification volumes in the aortic arch, landing zone and left ventricle were not associated with the increase in white matter hyperintensity volume. In 72% of the patients new chronic white matter hyperintensities developed 3 months after TAVI, with a median increase of 27%. A higher calcification volume in the AV was associated with a larger increase in the white matter hyperintensity volume. These findings show the potential for automated AV calcium screening as an imaging biomarker to predict chronic silent brain infarctions.

**Electronic supplementary material:**

The online version of this article (10.1007/s10554-019-01663-0) contains supplementary material, which is available to authorized users.

## Background

Transcatheter aortic valve implantation (TAVI) has been established as an effective treatment of severe aortic valve (AV) stenosis, reducing both clinical symptoms and mortality. Nevertheless, one of the most feared complications, namely the occurrence of stroke, is still observed in 2.4% of the patients undergoing TAVI [1]. This complication is observed despite the expansion to low-risk populations, the increase in operator experience and the development of improved valve-systems [2]. The cerebral emboli during TAVI are attributed to the dislodgement of aortic arch calcifications during the advancement of the catheter through the orifice and the expansion of the implanted valve crushing the calcified native valve [3]. Our previous research showed that stroke after TAVI is associated with poor outcomes, including a sixfold increase of procedural mortality [1]. Nevertheless, clinically overt strokes are only the tip of the iceberg, since shortly after TAVI acute silent cerebral infarctions are visible with diffusion-weighted magnetic resonance imaging (DW-MRI) in up to 78% of the patients [4]. Unfortunately, currently the majority of the TAVI-induced cerebral emboli remains unpredictable.

Cardiac computed tomography angiography (CTA) scans are part of the routine pre-TAVI work-up and may be used to measure the local calcification burden in the area of the AV and arch [5]. This is confirmed by a previous study that showed that a large plaque volume in the AV prior to TAVI predicted the occurrence of acute silent brain infarctions several days after TAVI [6]. Despite a high increase of acute silent brain infarctions, these lesions have as yet not been related to long-term cognitive deterioration [7]. Previous research showed that only a proportion of the acute silent brain infarctions, as detected with DW-MRI several days after TAVI, proceeded into chronic loco-regional remnants, which can be identified as white matter hyperintensities on fluid attenuation inversion recovery (FLAIR) MRI sequence, several months after TAVI [7].

In contrast to acute silent brain infarctions, chronic white matter hyperintensities that emerged after the procedural phase were associated with a decrease in cognitive functioning during long-term follow-up [7]. Moreover, in the general elderly population white matter hyperintensities are associated with cognitive impairment, a twofold higher risk of dementia and a threefold higher risk of stroke [8]. TAVI has been developed to improve quality of life, however the increase in TAVI-associated white matter hyperintensities may evoke cognitive deterioration and subsequently compromise self-reliance and increase healthcare costs. If it could be predicted which patients are at risk for developing chronic white matter hyperintensities after TAVI, these high-risk patients could be protected by using additional procedural protective methods, such as cerebral protections devices.

We investigated for the first time to which extent the calcification volume of the native *aortic valve*, *aortic arch*, *landing zone and left ventricle* measured with cardiac CTA, can be used to predict the increase in chronic white matter hyperintensities after TAVI*, indicating new chronic brain infarctions*.

## Methods

### Study population and trial design

The current study is a single center, prospective trial, initiated and managed by the Heart Center of the Amsterdam University Medical Center (UMC), a high volume tertiary cardiac care and TAVI center. Patients with severe AV stenosis planned to undergo TAVI of a native valve were eligible for inclusion in the trial. Exclusion criteria for trial participation were known structural brain disease (including a history of stroke or transient ischemic attacks) or presence of an MRI contra-indication. The decision for TAVI treatment was made by our multidisciplinary TAVI team consisting of a cardiac surgeon, an interventional-cardiologist, a radiologist, a geriatric internist and a dedicated nurse-practitioner. Moreover, the dedicated multidisciplinary TAVI-team selected the access route and the valve size, all according to the most recent guidelines [5]. The transfemoral approach was the default access option, alternatively, in patients with severe peripheral artery disease the transaortic route was used. The study protocol was approved by the Institutional Review Boards of the Academic Medical Center and all participants provided written informed consent. The current study is registered at the Netherlands Trial Register (NL7495).

### Endpoints

**Fig. 1:**
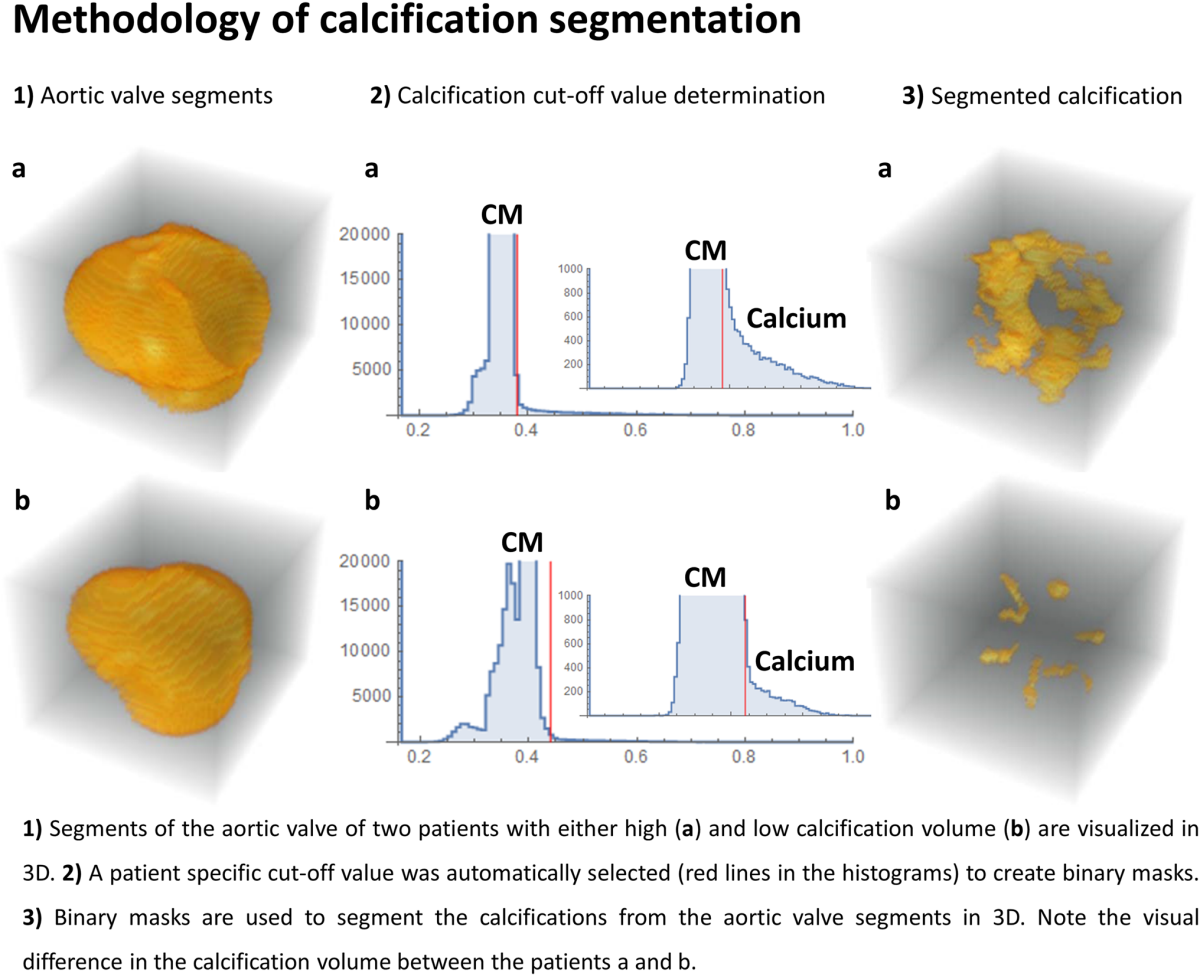
Methodology of calcification segmentation

The main objectives of the study were to determine the distribution of calcification volumes (mm^3^) using CTA in four prespecified sections of the heart and aortic arch of patients undergoing TAVI (Fig. 1) and to determine whether an association exists between the prespecified regional calcification volumes (mm^3^) and the increase in FLAIR-MRI determined cerebral white matter hyperintensity volume (Δ% from baseline mL), 3 months after TAVI. This relative increase (Δ%) rather than the actual increase (ΔmL) was chosen since we expected large variations in baseline white matter hyperintensity volumes between patients and baseline white matter hyperintensity volume is known to predict the actual increase in volume over time (ΔmL) [9]. Hence, by selecting the relative increase (Δ%) we investigated the influence of the TAVI-procedure rather than the baseline patient risk. The secondary objective was to determine clinical (patient) predictors of both the calcification volume (mm^3^) and of actual increase in new white matter hyperintensities (mL).

### Cerebral MRI and cardiac CTA acquisition and image analyses

**Fig. 2:**
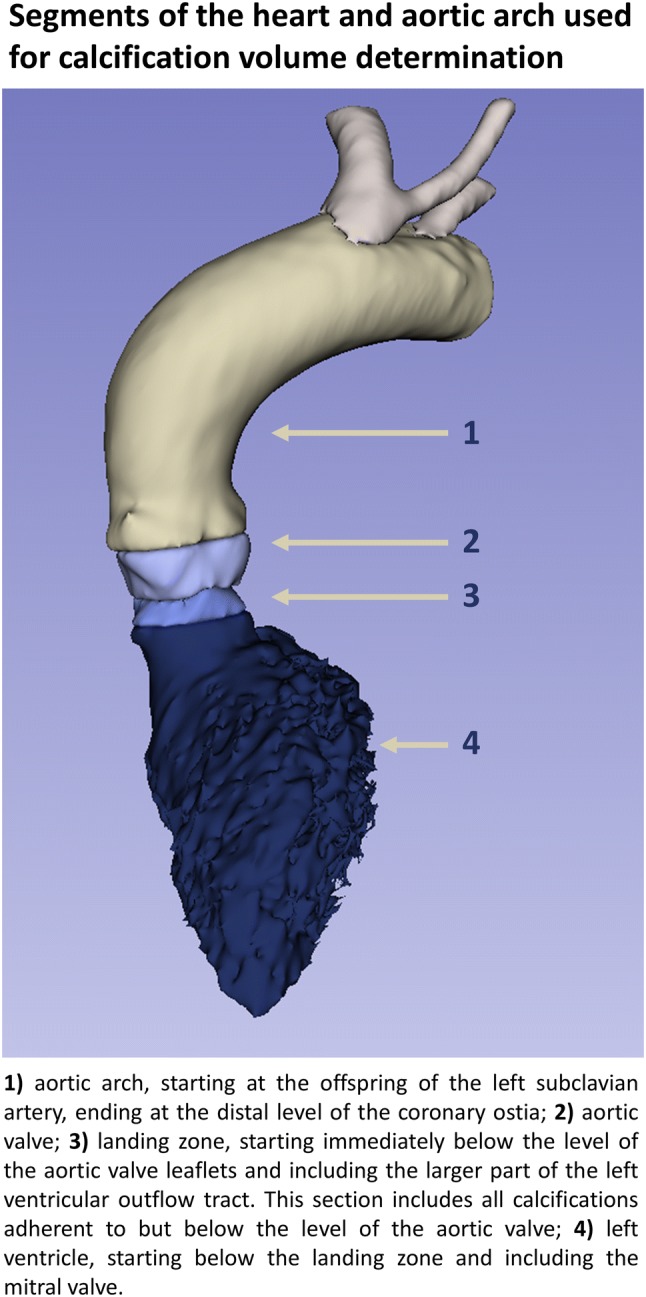
Segments of the heart and aortic arch used for calcification volume determination

A detailed description of the image acquisition and analyses is provided as supplementary text with corresponding table and figure (Fig. 1; Supplementary text 1 and Table 1). In brief, the included patients underwent cerebral MRI evaluation of white matter hyperintensities using a FLAIR sequence at a 3-T MRI scanner at baseline ( < 24 h prior to the TAVI procedure) and 3 months after the TAVI. The FLAIR sequence was used because of its sensitivity to measure white matter hyperintensities, appearing as lesions with increased brightness. White matter hyperintensities were quantified using an automated segmentation method. CTA scans were acquired in all patients as part of the routine pre-TAVI work-up and used to measure the calcification volumes in the selected segments (Fig. 2). In these CTA scans, patient-specific cut-off values for calcifications were automatically detected to minimize differences in calcification volume measurements between patients with different CTA setting [tube voltage (kV)] and different contrast delivery protocol. These cut-off values were based on the distribution of the amount of pixels with pixel-values, corresponding with the primary components of the segments (calcifications, contrast material and soft-tissue) (Fig. 1).

**Table 1:** Baseline patient and procedural characteristics

	Study population (n = 36)
Demographics	
Age (years)	78.7 ± 4.5
Female gender	22 (61%)
Body mass index (kg/m^2^)	29.8 ± 7.4
Medical history	
Previous myocardial infarction	6 (17%)
Previous PCI	10 (28%)
Previous CABG	3 (8%)
Diabetes mellitus	10 (28%)
Hypertension	21 (58%)
Dyslipidemia	6 (17%)
History of coronary artery disease	14 (39%)
Atrial fibrillation	16 (44%)
Glomerular filtration rate < 30 mL/min/1.73 m^2^	3 (8%)
NT-proBNP (ng/L)	882 (361–2775)
New York Heart Association (NYHA) class III or IV	22 (61%)
Risk scores	
EuroSCORE II (%)	1.9 (1.5–3.4)
STS-PROM mortality (%)	2.8 (1.9–3.7)
Echocardiographic characteristics	
Aortic max gradient (mmHg)	68 ± 24
Aortic mean gradient (mmHg)	43 ± 17
Aortic valve area (cm^2^)	0.74 ± 0.16
Procedural details	
Transfemoral access	33 (92%)
Transaortic access	3 (8%)
Edwards SAPIEN 3	35 (97%)
Direct flow	1 (3%)
Medication at discharge	
Single antiplatelet therapy	1 (3%)
Dual antiplatelet therapy	18 (50%)
Single antiplatelet therapy + anticoagulation therapy (VKA/NOAC)	12 (33%)
Anticoagulation therapy only (VKA/NOAC)	5 (14%)
Statin	24 (67%)

Values are mean ( ± SD), n (%) or median (interquartile range)

*VKA* vitamin K antagonist, *NOAC* novel oral anticoagulant

### Statistical analysis

Continuous values of patient and MRI characteristics, including calcification volumes per segment and white matter hyperintensity volumes were tested for normal distribution and are reported as mean ± SD or median with interquartile ranges (Q_1_–Q_3_ 25–75th percentile) where appropriate. Accordingly, depending on the distribution of the data, either the independent t-test or Mann–Whitney U test determined differences between groups. Categorical variables are presented as frequencies and percentages, differences between groups were tested with Chi-square. Moreover, the calcification volumes per segment (mm^3^) were explored as predictors of the relative increase of white matter hyperintensities (Δ%) using linear regression. In a secondary analysis, to correct for inter-individual variance in intracranial volumes, the white matter hyperintensity volume (mL) was normalized for intracranial volume (mL). Moreover, baseline patient characteristics were explored by linear regression as predictors of either the baseline calcification volume per segment (mm^3^) or the increase of white matter hyperintensities (ΔmL). Potential clinical predictors were tested in a univariate model, and if p < 0.05, simultaneously entered to create a multiple regression model. The results of all regression models are reported as beta coefficients (B) and standard errors (SEs). All statistical tests were two-tailed, and a value of p < 0.05 was considered to indicate a statistically significant difference. Calculations were generated using SPSS software (version 25.0 for Windows, SPSS, Inc., Chicago, Illinois).

## Results

### Baseline patient characteristics

Between June 2016 and November 2017, a total of 48 patients with severe AV stenosis in the work-up for TAVI underwent baseline assessment of white matter hyperintensity volume. Twelve patients (25%) did not undergo follow-up cerebral MRI 3 months after the procedure related to either post-TAVI pacemaker implantation (n = 6) or refusal of MRI follow-up (n = 6). Accordingly, the final study population included 36 TAVI patients with complete MRI follow-up. Baseline demographic and clinical characteristics of the patients are provided in Table 1. Mean age was 78.7 ± 4.5 years with the majority (61%) female. The Society of Thoracic Surgeons (STS) median score of 2.8% (Q_1_–Q_3_ 1.9–3.7%) indicated this was a low-risk TAVI population. Nevertheless, many patients had comorbidities including diabetes (28%), atrial fibrillation (44%) and a history of coronary artery disease (39%). The majority of the patients underwent transfemoral TAVI (92%) with a balloon-expandable Edwards SAPIEN 3 valve (97%). Following TAVI, 50% of the patients were treated with dual antiplatelet therapy (DAPT) and 33% received single antiplatelet therapy (SAPT) in combination with anticoagulant therapy. Patients not undergoing MRI follow-up had a higher median EuroSCORE II (3.4%, Q_1_–Q_3_ 2.0–4.6%, vs. 1.9%, Q_1_–Q_3_ 1.5–3.5%, p = 0.03) and a larger AV area (0.91 ± 0.18 cm^2^ vs. 0.74 ± 0.16 cm^2^ vs., p = 0.004) prior to TAVI^.^(Supplementary Table 2).

### Cardiac and aortic calcification volumes

**Fig. 3:**
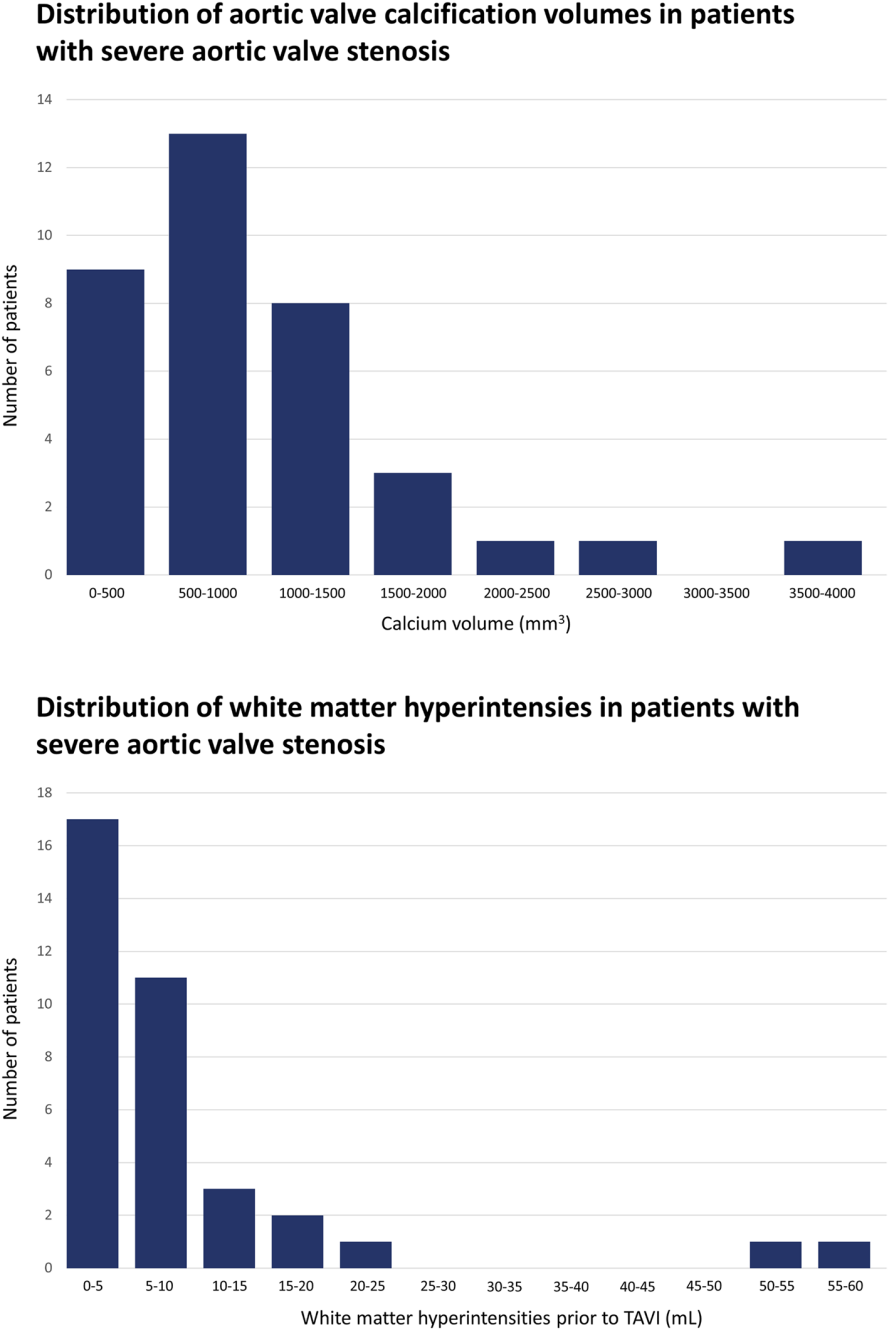
Distribution of aortic valve calcification volumes and white matter hyperintensities in patients with severe aortic valve stenosis

**Fig. 4:**
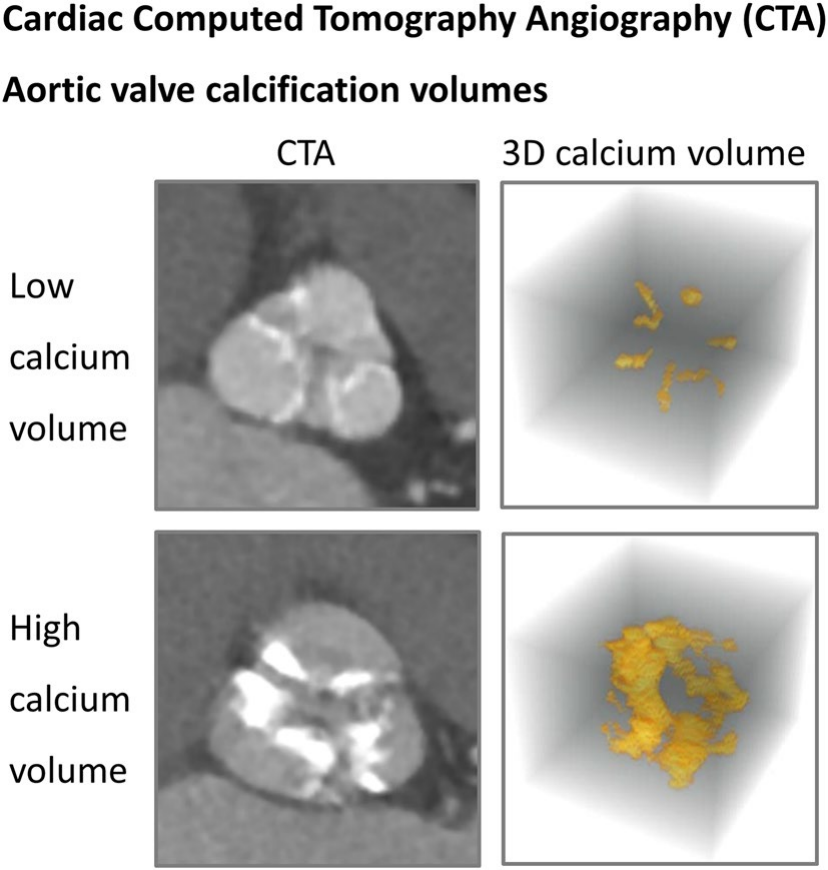
Cardiac computed tomography angiography (CTA) aortic valve calcification volumes

Out of the four prespecified segments median calcification volumes were the highest in the AV (692 mm^3^, Q_1_–Q_3_ 482–1297 mm^3^) and the aortic arch (633 mm^3^, Q_1_–Q_3_ 213–1727 mm^3^). The distribution of the calcification volumes in the AV are displayed in Fig. 3. A CTA example of a low versus a high calcification volume of the AV is shown in Fig. 4. In multiple regression analysis, AV calcification volumes were larger in patients with a larger (CT-determined) annular area, with a 3.8 mm^3^ (SE 1.2, p = 0.004) increase of AV calcification volume per every additional mm^2^ of annular area (Supplemental Table 3). Moreover, patients with a higher mean gradient had higher AV calcification volumes, with a 15.9 mm^3^ (SE 4.9, p = 0.003) increase per every additional mmHg mean gradient increase. The majority of patients with severe AV stenosis (82%) also had some calcifications in the left ventricle. These calcifications were generally located around the mitral valve and had a lower volume than the AV and arch (164 mm^3^, Q_1_–Q_3_ 19–1332 mm^3^). Finally, 63% of the patients had calcifications in the landing zone, with a relatively low calcification volume compared with the other segments (16 mm^3^, Q_1_–Q_3_ 0–68 mm^3^).

### Cerebral white matter hyperintensity volume

**Fig. 5:**
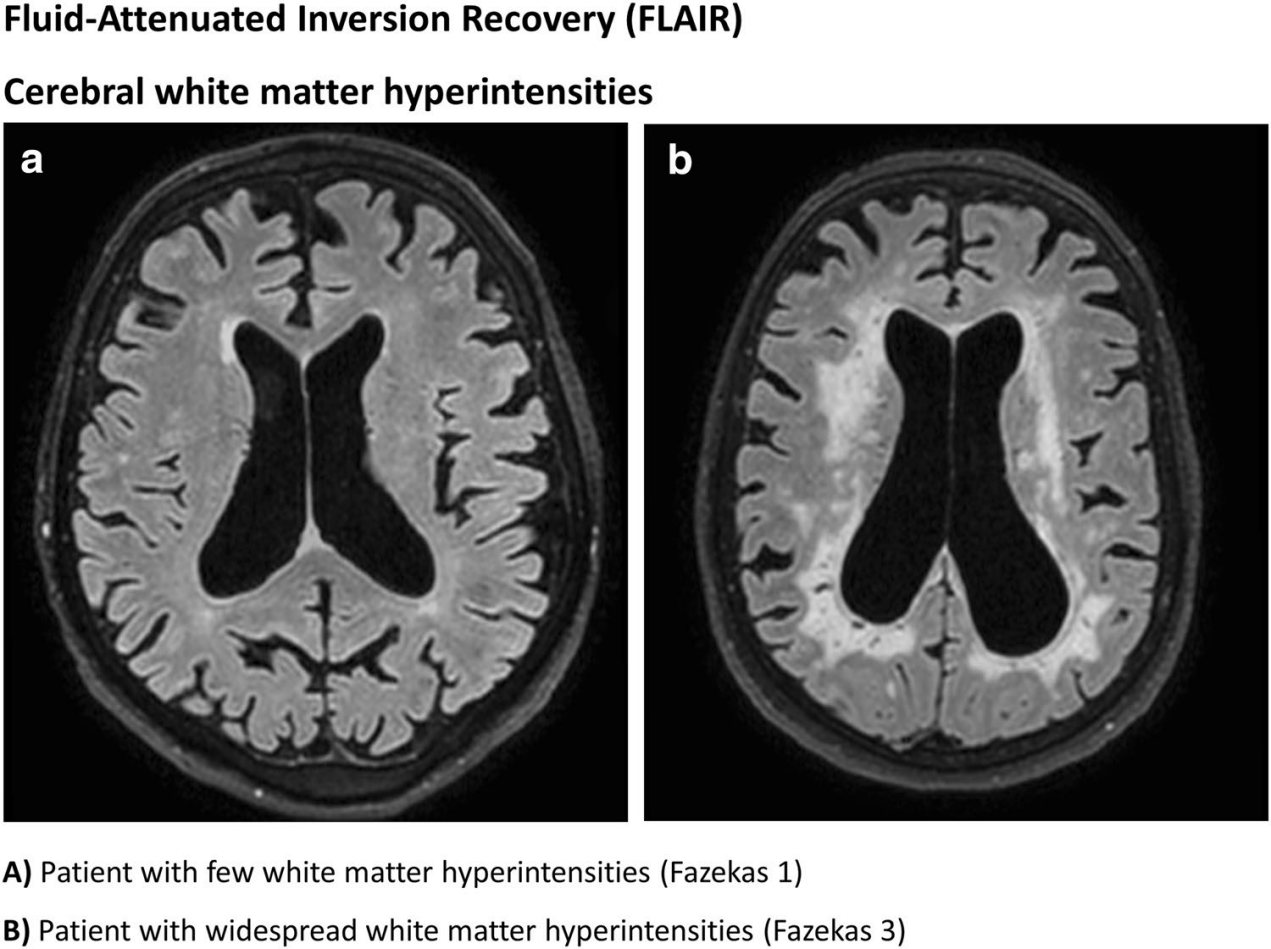
Fluid-attenuated inversion recovery (FLAIR) cerebral white matter hyperintensities

**Fig. 6:**
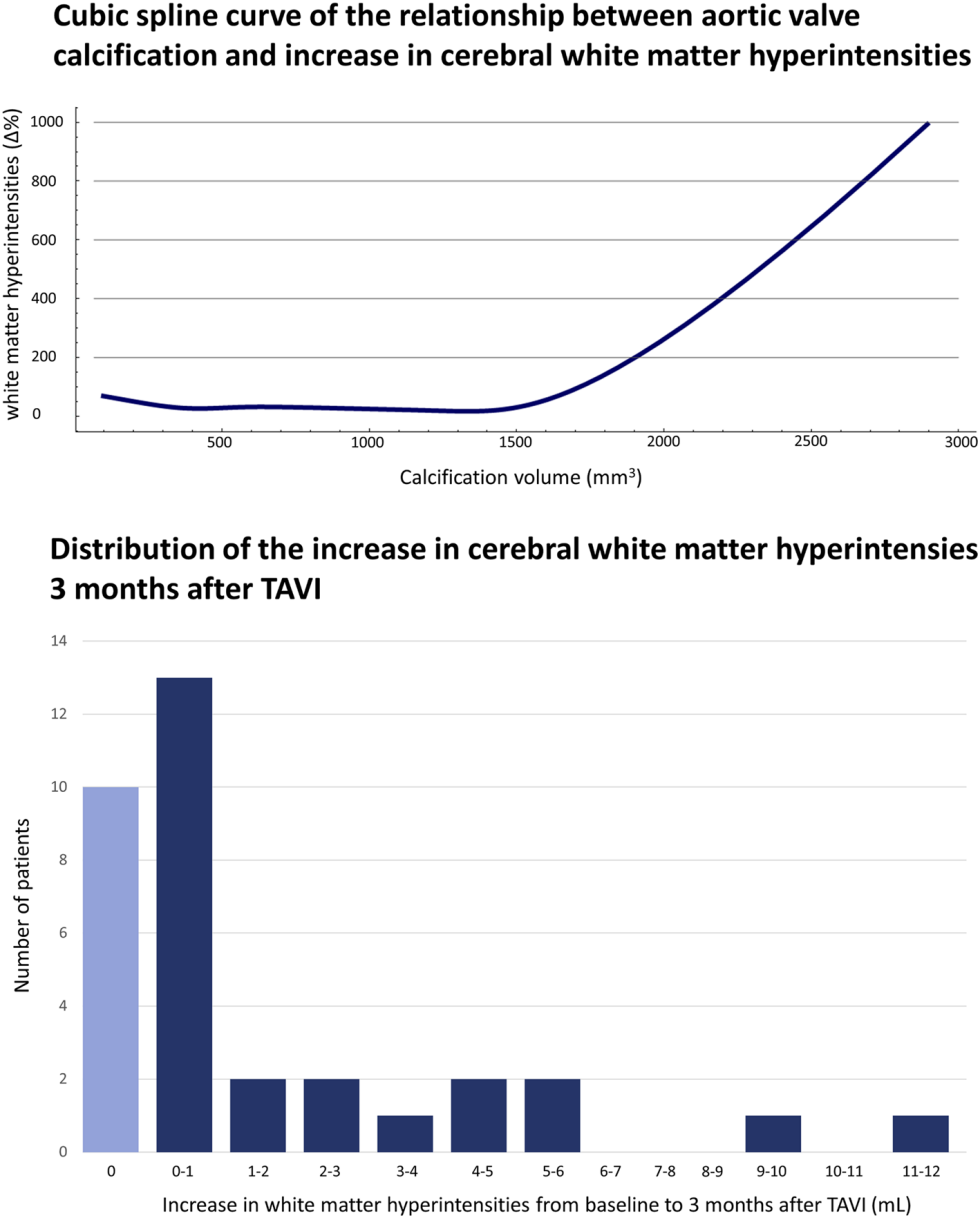
Cubic spline curve of the relationship between aortic valve calcification and increase in cerebral white matter hyperintensities. Distribution of the increase in cerebral white matter hyperintensities 3 months after TAVI

All patients with severe AV stenosis had white matter hyperintensities prior to TAVI, with a median volume of 5.1 mL (Q_1_–Q_3_ 2.1–8.9 mL), this was 0.4% of the total intracranial volume (Q_1_–Q_3_ 0.2–0.7%). The volume of baseline white matter hyperintensities varied considerably between patients, the distribution is given in Fig. 3. Figure 5 shows two patients with few versus widespread white matter hyperintensities. Patients with versus without pre-existing atrial fibrillation had a more than twofold higher baseline white matter hyperintensity volume (8.6 mL, Q_1_–Q_3_ 4.2–13.9 mL vs. 3.7 mL, Q_1_–Q_3_ 1.4–6.0 mL, p = 0.03). None of the patients experienced clinical overt stroke during the follow-up period. Nevertheless, in 26 patients (72%) the white matter hyperintensity volume increased 3 months after TAVI. The median increase in these patients was 1.1 mL (Q_1_–Q_3_ 0.3–4.6 mL), corresponding with a 27% increase from baseline (Q_1_–Q_3_ 7–104%), the distribution of the increase in white matter hyperintensity volume is shown in Fig. 6. The calcification volume of the AV predicted the relative increase of white matter hyperintensity volume at follow-up (Δ%), with a 35.2% increase per 100 mm^3^ of native AV calcification volume (SE 8.5, p < 0.001). The relationship between AV calcification volume and the increase in white matter hyperintensities is displayed in Fig. 6. In contrast, the calcification volume of the AV was not associated with the volume of baseline white matter hyperintensities (B 0.0, SE 0.0, p = 0.87). Furthermore, the calcification volume in the aortic arch (B 3.0%, SE 3.7, p = 0.42), landing zone (B − 31.9%, SE 79.5, p = 0.69) and left ventricle (B − 3.9%, SE 6.5, p = 0.55) did not predict the relative increase in cerebral white matter hyperintensity volume. There was a considerable variation in intracranial volumes between patients, with a median volume of 1352 mL (Q_1_–Q_3_ 1205–1468 mL) at baseline. Correction for intracranial volumes resulted in similar outcomes, with the AV calcification volume remaining a significant predictor of the relative increase in white matter hyperintensity volume (p < 0.001). In multiple regression analysis, no clinical factors other than the calcification burden of the AV predicted the increase of white matter hyperintensity volume (Supplemental Table 4).

## Discussion

### Main findings

This is the first study to address the relationship between calcification volumes of the heart and aortic arch prior to TAVI and chronic brain infarctions following TAVI. The main findings were that in patients with severe AV stenosis a large variation exists in CTA assessed calcification volumes of the AV. Likewise, the variation in baseline white matter hyperintensity volumes is considerable, with a twofold higher volume in patients with history of atrial fibrillation. Cerebral white matter hyperintensity volume increased in the majority of patients (72%) during follow-up. A higher volume of AV calcification volume pre-TAVI was associated with a larger increase of white matter hyperintensity volume during follow-up, indicating chronic brain infarctions. Altogether, these findings show the potential for automated AV calcium screening as an imaging biomarker to predict chronic silent brain infarctions in patients undergoing TAVI.

### Whiter matter hyperintensities after TAVI

The findings of the current study emphasize that the TAVI procedure induces a chronic cerebral embolic burden in an already frail population. In the current low-risk TAVI population, 7 out of 10 patients undergoing TAVI had an increase in white matter hyperintensity volume, with a median increase of 1.1 mL (Q_1_–Q_3_ 0.3–4.6 mL) in the course of 3 months. In comparison, the mean white matter hyperintensity volume increased with the same volume (1.1 ± 1.8 mL) in the course of 4 years in a population-based study in healthy subjects with a comparable mean age of 79 years [10]. Further emphasizing the embolic burden in a portion of the patients, we found that half of the patients had an increase of new white matter hyperintensities < 1 mL, whereas the other half had an increase of 2–12 mL. In population based studies, white matter hyperintensities were related to impaired neuropsychological functioning across all domains including memory, verbal fluency and mental slowing [11].

Similar to findings of the current study, a previous study found that the calcification volume of the AV, measured with the Agatston calcium score, predicted the number of solid cerebral emboli during the TAVI procedure measured with transcranial Doppler [12]. The highest number of cerebral embolizations takes place during the phase of the valve positioning, indicating that calcifications dislocate during valve placement. Similarly, the current results showed that the AV volume predicted the relative increase in white matter hyperintensity volume, rather than the calcification volume in the aortic arch, landing zone and left ventricle. This is despite the fact that large sized catheters are approached through the aortic arch with substantial calcification volumes in almost all patients. This significant role of AV calcifications in cerebral embolizations during the TAVI procedure is underlined by clinical studies indicating that patients with a smaller AV area, higher pre-TAVI AV gradient, increased aortic arch atheroma and/or severe aortic calcification, all indicating a higher calcification burden, are more likely to experience early post-procedural cerebrovascular events [1, 13–16]. Moreover, not the calcification volume itself, but the plaque volume in the AV of a lower density than calcifications (50–130 HU), matching fibrous tissue, was shown to be associated with the occurrence of new DW-MRI lesions short after TAVI [6]. However, studies in TAVI populations did not find a relationship between occurrence of these new DW-MRI lesions after TAVI and (long-term) neurocognitive decline [17]. A mismatch between the presence of acute ischemic lesions detected with DW-MRI and the absence of chronic white matter hyperintensities detected with FLAIR, can be applied to identify salvageable brain areas [18]. In contrast to acute DW-MRI lesions, there is an association between long-term white matter hyperintensities in TAVI patients and a clear trend of cognitive decline, indicating that not all acute silent infarctions continue to be chronic brain infarctions [7]. Accordingly, in contrast to the DW-MRI study, the findings of the current study highlight the relationship between calcification volume in patients with AV stenosis and the increased risk of new white matter hyperintensities after TAVI, which will potentially induce long-term cognitive deterioration.

### The risk of atrial fibrillation in TAVI patients

The reported prevalence of pre-existing atrial fibrillation in TAVI patients ranges from 16 to 51% [19]. An interesting finding of the current study was that patients with a history of atrial fibrillation had a more than twofold higher volume of baseline white matter hyperintensity volume before TAVI compared without pre-existing atrial fibrillation. This finding is in accordance with data from a large population-based study [20]. Patients with atrial fibrillation are at heightened risk of cognitive decline and dementia, even in the absence of a medical history of past stroke [13]. Moreover, compared with healthy control subjects, patients with atrial fibrillation have a lower cerebral perfusion and impaired cerebrovascular reactivity indicative of a limited cerebral vascular reserve [14]. These findings emphasize the risk of atrial fibrillation on cognitive functioning in patients with AV stenosis.

### Reduction of new white matter hyperintensities after TAVI

Unfortunately, reliable clinical characteristics that predict the risk of (silent) brain infarctions after TAVI are lacking in the existing literature. We believe the findings from the current study, at the background of previous studies, indicate that the routinely performed CTA during pre-TAVI work-up could identify patients at risk for TAVI induced chronic silent brain infarctions. Identification of significant ascending aortic atheroma, using preoperative CT, in patients undergoing cardiac surgery, followed by tailored alternative operative strategies led to a threefold lower stroke rate compared with patients who did not undergo aortic atheroma identification with CT (p = 0.05) [15]. In a similar way, the AV calcification volume, measured with automated algorithms in CTA’s during the pre-TAVI work-up could assess the risk of cerebral embolization during TAVI and identify patients that could benefit from expensive cerebral protection devices [16].

### Limitations

The current study did not include a control population, that did not undergo TAVI. Moreover, the current study included a low-risk population, whereas the calcification burden may be even higher in intermediate and high-risk populations. Subsequently, a higher calcification burden may be associated with a higher volume of new white matter hyperintensities after TAVI. Similarly, the current study included only balloon-expandable valves, even though self-expandable valves have been associated with a higher stroke rate [21]. Balloon-expandable valves are implanted rather rapidly, in contrast, self-expandable valves are implanted stepwise and slowly and allow repositioning before the final implantation. Therefore, we cannot exclude a relation between the calcification volume in the ascending aorta or left ventricular outflow trajectory and new white matter hyperintensities in patients treated with self-expandable valves. Furthermore, were not able to use the commonly used Agatston calcium score as this has not been validated in contrast enhanced cardiac CTA scans. Non-contrast CT scans of the heart were not part of the TAVI work-up. Moreover, white matter hyperintensities not only include chronic brain infarctions but also regions of small vessel disease. The former is characterized by a rather acute increase of volume, whereas the latter is characterized by a slow gradual increase. Therefore, we believe that by selecting the relative increase (Δ%) over the course of a short period of 3 months as the endpoint, the measured outcome mainly detects new infarctions rather than small vessel disease. Finally, cognitive functioning tests were not performed in the current study and relationships between the AV calcification volume, the volume of new white matter hyperintensities and cognitive functioning cannot be confirmed. Even though various studies reported a relationship between the increase in white matter hyperintensities and neuropsychological functioning, there is currently limited evidence reporting an increase in white matter hyperintensity volumes is associated with neurocognitive impairment [22, 23].

## Conclusions

In a low-risk population with severe AV stenosis, all patients had valve calcifications prior to TAVI, with a large variation in calcification volumes between patients. The majority of the patients (72%) had new cerebral white matter hyperintensities 3 months after TAVI, with a median increase of 27%. A larger calcification volume in the AV, but not in the aortic wall, landing zone and left ventricle prior to TAVI was associated with a higher increase in cerebral white matter hyperintensity volume after TAVI, indicating new chronic brain infarctions.

## Electronic supplementary material

Below is the link to the electronic supplementary material.

Supplementary file1 (DOCX 467 kb)
