# Supplementary material for: Aortic valve calcification volumes and chronic brain infarctions in patients undergoing transcatheter aortic valve implantation
Source: Int J Cardiovasc Imaging. 2019 Jul 16;35(11):2123–33. doi: 10.1007/s10554-019-01663-0 (PMC6805808; doi:10.1007/s10554-019-01663-0)
Supplement: Supplementary file 1 — Supplementary file1 (DOCX 467 kb) [file 10554_2019_1663_MOESM1_ESM.docx]

### **Supplementary text 1**

### Coronary computed tomographic image acquisition and analysis

### Cardiac CTA acquisition was performed on a third-generation dual-source CT scanner in the majority of the patients (SOMATOM Force, Siemens Healthcare, Erlangen, Germany) and in the minority of patients on a second generation dual-source CT scanner (SOMATOM Definition AS, Siemens Healthcare, Erlangen, Germany) or a single source CT scanner (Phillips iCT 256 Brilliance, Phillips Healthcare, Best, Netherlands). Technical specifications are listed in supplementary table 1. Retrospective ECG-gated acquisition was performed in all patients. Tube voltage ranged from 70-140 kV and tube current was adjusted according to individual body habitus. During cardiac CTA acquisition, 80-110ml (4.5-5.5 ml/s) iodinated contrast (Ultravist 300: iopromide 300 mg I/ml, Bayer Healthcare Pharmaceuticals, Whippany, USA) was injected into an antecubital vein and followed by a 40 ml saline flush (5 ml/s). The images were reconstructed with a slice thickness of 1.5 mm and an increment of 1.5 mm using iterative reconstruction.

**Supplemental table 1.** CT-scanner specifications and image acquisition with contrast delivery details

|  | **Number of patients** | **Detector rows**  **(slices)** | **Rot.**  **Time**  **(ms/rot)** | **Tube voltage settings**  **(kV)** | **Tube charge settings**  **(mAs)** | **Contrast conc.**  **(mg I/ml)** | **Contrast**  **volume**  **(ml)** |
| --- | --- | --- | --- | --- | --- | --- | --- |
| Siemens SOMATOM Force | 31 | 2 x 96  (2 x 192) | 250 | 70-120 | 75-330 | 300 | 80 |
| Siemens SOMATOM Definition AS+ | 3 | 64  (128) | 300 | 100 | 75-175 | 300 | 110 |
| Phillips iCT 256 Brilliance | 2 | 128  (256) | 270 | 100-140 | 130-600 | 300 | 80-100 |

### The cardiac and aortic regions were manually labelled using version 4.8.1 of 3D slicer (1,2). The labelled regions were: **1)** aortic arch, starting at the offspring of the left subclavian artery, ending at the distal level of the coronary ostia; **2)** aortic valve: **3)** landing zone, starting immediately below the level of the aortic valve leaflets and including the larger part of the left ventricular outflow tract. This section included all calcifications adherent to but below the level of the aortic valve leaflets; **4)** left ventricle, starting below the landing zone and including the mitral valve. Wolfram Mathematica (Version 11.3; Wolfram Research, Inc., Champaign, Il, USA) was used for segmentation and post processing.

### Since intravascular attenuation and the attenuation of calcium in cardiac CTA are susceptible to changes in contrast delivery and tube voltage settings, we were not able to select a single cut-off value for the semi-automatic segmentation of calcium. To overcome this problem, pixel value mapping was applied per patient in the combined segmented 3D volumes to identify the main components (contrast material and calcium). A patient specific cut-off value was automatically selected, based on these main components and used to create binary masks. These masks, containing all pixels with a pixel value higher than the selected cut-off value (a pixel value higher than contrast material), were used to segment the calcium from each individual segment. Calcium volume was calculated by multiplying the pixels by the pixel dimensions. **Figure 1** in the manuscript shows a systematical representation of the described method for two aortic valve segments of patient with low and high calcium volume.

**Supplementary figure 1**


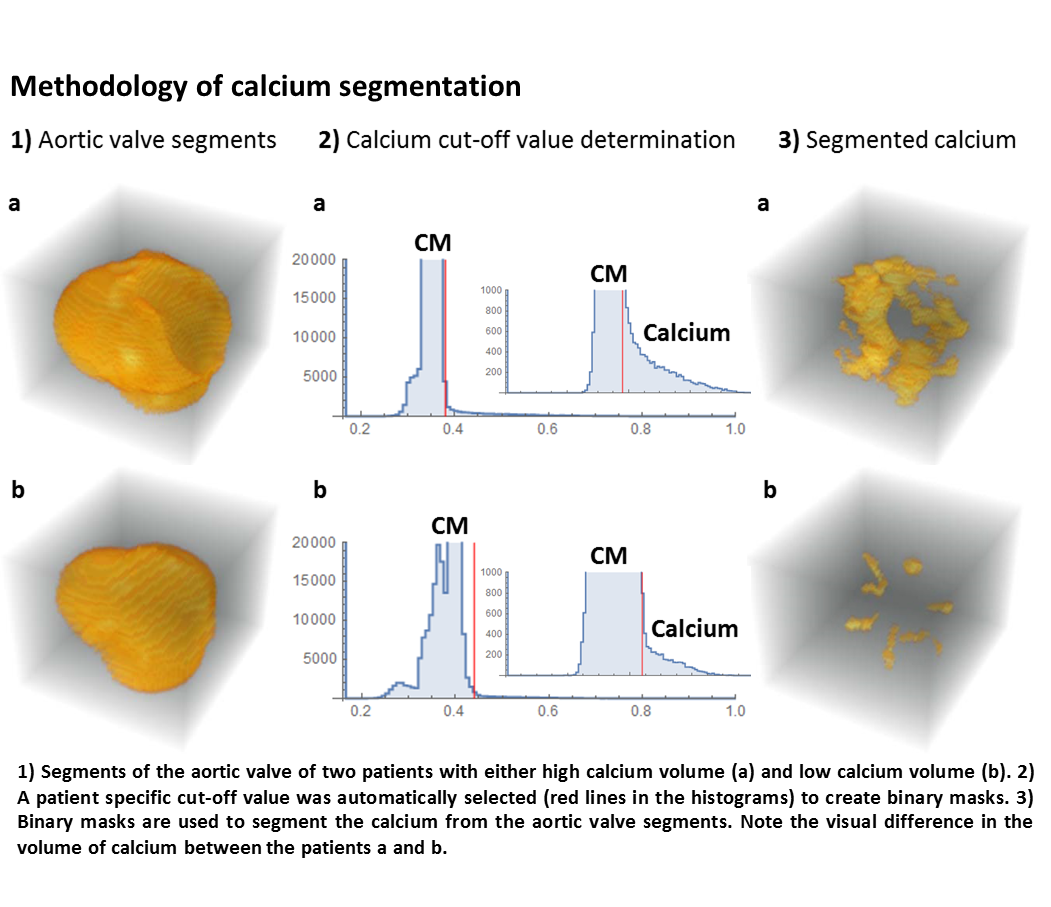


### White matter hyperintensity volume measurement

Brain MRI scans were obtained at baseline (within 24 hours prior to TAVI) and at 3 months follow-up. Participants were all scanned on the same 3-Tesla Philips Ingenia MRI-scanner (Best, the Netherlands) both during baseline and at follow-up (C.B.L.M). The scan quality was reviewed visually by a neuroradiologist. White matter hyperintensities were automatically segmented from the fluid attenuation inversion recovery (FLAIR) scans using a method based on (3). In addition, an automated brain segmentation method was applied to the T1-weighted scans. Both segmentation methods were developed by Quantib B.V. From these segmentations, we computed the intracranial volume and the volume of white matter hyperintensities (mL). White matter fraction was computed by dividing a patient’s white matter volume by their intracranial volume. The primary endpoint was the relative increase (Δ%) in white matter hyperintensities per patient from baseline to follow-up.

**Supplementary table 2. Baseline patient and procedural characteristics in study population vs lost to follow-up patients**

|  | **Total population**  (N=48) | **Study population**  (n=36) | **No follow-up** (n=12) | **p-value (*)** |
| --- | --- | --- | --- | --- |
| **Demographics**  Age (years)  Female gender  Body mass index (kg/m^2^) | 79.2 ± 5.0  26 (54%)  28.8 ± 6.8 | 78.7 ± 4.5  22 (61%)  29.8 ± 7.4 | 80.7 ± 6.1  4 (33%)  25.9 ± 3.8 | 0.24  0.09  0.09 |
| **Medical history**  Previous myocardial infarction  Previous PCI  Previous CABG  Diabetes mellitus  Hypertension  Dyslipidemia  History of coronary artery disease  Atrial fibrillation  GFR < 30 ml/min/1.73m^2^  NT-proBNP  NYHA class III or IV | 8 (17%)  12 (25%)  4 (8%)  13 (27%)  27 (56%)  11 (23%)  18 (38%)  21 (44%)  4 (8%)  1065 (440-2775)  27 (56%) | 6 (17%)  10 (28%)  3 (8%)  10 (28%)  21 (58%)  6 (17%)  14 (39%)  16 (44%)  3 (8%)  882 (361-2775)  22 (61%) | 2 (17%)  2 (17%)  1 (8%)  3 (25%)  6 (50%)  5 (42%)  4 (33%)  5 (42%)  1 (8%)  1088 (627-4118)  5 (42%) | 1.00  0.44  1.00  0.85  0.61  0.07  0.73  0.87  1.00  0.18  0.24 |
| **Risk scores**  EuroSCORE II (%)  STS-PROM mortality (%) | 2.2 (1.6-3.5)  2.8 (1.9-3.7) | 1.9 (1.5-3.4)  2.8 (1.9-3.7) | 3.4 (2.0-4.7)  2.7 (1.9-4.1) | 0.03  0.71 |
| **Echocardiographic characteristics**  Aortic max gradient (mmHg)  Aortic mean gradient (mmHg)  Aortic valve area (cm^2^) | 65 ± 24  41 ± 16  0.78 ± 0.18 | 68 ± 24  43 ± 17  0.74 ± 0.16 | 56 ± 23  34 ± 12  0.91 ± 0.18 | 0.11  0.07  0.004 |
| **Procedural details**  Transfemoral access  Transaortic access  Transapical access  Edwards SAPIEN 3  Direct Flow | 44 (92%)  3 (6%)  1 (2%)  47 (98%)  1 (2%) | 33 (92%)  3 (8%)  0 (-)  35 (97%)  1 (3%) | 11 (92%)  0 (-)  1 (8%)  12 (100%)  0 (-) | 0.30  0.30  0.08  0.56  0.56 |
| **Medication at discharge**  SAPT  DAPT  SAPT + VKA/NOAC  VKA/NOAC  Statin | 4 (8%)  21 (44%)  17 (35%)  6 (13%)  33 (69%) | 1 (3%)  18 (50%)  12 (33%)  5 (14%)  24 (67%) | 3 (25%)  3 (25%)  5 (42%)  1 (8%)  9 (75%) | 0.02  0.13  0.60  0.61  0.59 |

*Values are mean (± SD), n (%) or median (interquartile range). PCI = percutaneous coronary intervention, CABG = coronary artery bypass grafting, GFR = glomerular filtration rate, NT-ProBNP = N-terminal prohormone of brain natriuretic peptide, NYHA = New-York Heart Association, EuroSCORE = European System for Cardiac Operative Risk Evaluation, STS-PROM = Society of Thoracic Surgeons Predicted Risk Of Mortality, SAPT= single anti-platelet therapy, DAPT= double anti-platelet therapy, VKA= vitamin K antagonist, NOAC=platelet therapy + VKA/NOAC. VKA = vitamin K antagonist. NOAC = novel oral anticoagulant.*

**Supplementary table 3. Univariate and multiple regression of calcium volume in the aortic valve**

|  | **Univariate, B (SE)** | **P-Value** | **Multiple, B (SE)** | **P-Value** |
| --- | --- | --- | --- | --- |
| **Demographics**  Age (per year increase)  Male gender  Body mass index (per kg/m^2^ increase)  Length (per cm increase) | 3.32 (29.39)  991.29 (208.25)  7.40 (17.96)  62.14 (13.15) | 0.91  <0.001  0.68  <0.001 | -  273.51 (247.86)  -  21.95 (14.17) | -  0.28  -  0.13 |
| **Medical history**  Previous myocardial infarction  Previous PCI  Diabetes mellitus  Hypertension  Dyslipidemia  History of coronary artery disease  Atrial fibrillation  GFR < 30 ml/min/1.73m^2^  (NYHA) class III or IV  Number of stents during pre-TAVI PCI (per stent) | -180.80 (350.28)  -327.46 (287.15)  -263.63 (289.08)  74.74 (265.51)  -238.60 (349.26)  126.35 (267.95)  230.29 (260.76)  307.64 (471.22)  171.12 (267.22)  541.00 (218.33) | 0.61  0.26  0.37  0.78  0.50  0.64  0.38  0.52  0.53  0.13 | -  -  -  -  -  -  -  -  -  - | -  -  -  -  -  -  -  -  -  -  - |
| **Risk scores**  EuroSCORE II (per % increase)  STS-PROM mortality (per % increase) | -63.50 (86.97)  -60.14 (73.77) | 0.47  0.42 | -  - | -  - |
| **Imaging characteristics**  Aortic max gradient (per mmHg increase)  Aortic mean gradient (per mmHg increase)  Aortic valve area (per 0.1 cm^2^ increase)  CT determined annular area (per mm^2^ increase) | 12.61 (5.18)  18.29 (7.58)  -89.57 (80.41)  5.88 (1.02) | 0.02  0.02  0.27  <0.001 | -  15.85 (4.86)  -  3.79 (1.20) | -  0.003  -  0.004 |

*B = the increase of aortic valve calcium volume (in mm^3^ with 95% with standard error. The max gradient was not used in the multiple regression prediction model since this is directly related to the mean gradient.*

**Supplementary table 4. Univariate regression analysis of increase in cerebral white matter hyperintensities (ΔmL)**

|  | **Univariate, B (SE)** | **P-Value** |
| --- | --- | --- |
| **Demographics**  Age (per year increase)  Male gender  Body mass index (per kg/m^2^ increase) | -0.05 (0.11)  -0.28 (1.00)  0.04 (0.07) | 0.63  0.78  0.59 |
| **Medical history**  Previous myocardial infarction  Previous PCI  Previous CABG  Diabetes mellitus  Hypertension  Dyslipidemia  Current smoking  History of coronary artery disease  Atrial fibrillation  GFR < 30 ml/min/1.73m^2^  (NYHA) class III or IV | -0.05 (1.31)  -0.18 (1.09)  0.84 (1.75)  1.55 (1.05)  1.44 (0.96)  0.24 (1.30)  -0.10 (1.56)  -0.46 (1.00)  0.90 (0.97)  -0.37 (1.76)  -1.17 (0.98) | 0.97  0.87  0.64  0.15  0.14  0.86  0.95  0.65  0.36  0.84  0.24 |
| **Risk scores**  EuroSCORE II (per % increase)  STS-PROM mortality (per % increase) | 0.22 (0.32)  -0.12 (0.28) | 0.50  0.66 |
| **Echocardiographic characteristics**  Aortic max gradient (per mmHg increase)  Aortic mean gradient (per mmHg increase)  Aortic valve area (per 0.1 cm^2^ increase) | -0.02 (0.02)  -0.03 (0.03)  -0.59 (0.29) | 0.38  0.36  0.049 |

*B = the increase of cerebral white matter hyperintensities (in mL with standard error)*

**Supplementary references**

1. 2004 IEEE International Symposium on Biomedical Imaging: From Nano to Macro. IEEE Trans Med Imaging. 2004;

2. Fedorov A, Beichel R, Kalpathy-Cramer J, Finet J, Fillion-Robin JC, Pujol S, et al. 3D Slicer as an image computing platform for the Quantitative Imaging Network. Magn Reson Imaging. 2012;

3. de Boer R, Vrooman HA, van der Lijn F, Vernooij MW, Ikram MA, van der Lugt A, et al. White matter lesion extension to automatic brain tissue segmentation on MRI. Neuroimage. 2009;45(4):1151-61.
